# A Survey of Saudi General Practitioners on the Use of Thromboprophylaxis Guidelines and Risk Assessment Tools in Atrial Fibrillation

**DOI:** 10.3390/clinpract13020031

**Published:** 2023-02-23

**Authors:** Mohammed Ibrahim Alnami, Ali Mansoor Alsalim, Ruwaida Faisal Alhakeem, Bushra Abdulrahman Al-Somali, Haitham Ali Bahkali, Hanaa Ali Alhabshi, Hailah Talaq Alotaibi, Rahma Abdulrazzaq Alqallaf, Sheraz Ali

**Affiliations:** 1 Pharmaceutical Care Services, King Saud Medical City, Ministry of Health, Riyadh 11196, Saudi Arabia; 2 Menzies Institute for Medical Research, University of Tasmania, Hobart 7000, Australia

**Keywords:** thromboprophylaxis, atrial fibrillation, oral anticoagulants, stroke

## Abstract

Clinical practice guidelines advise patients with atrial fibrillation who are at risk for stroke to undergo thromboprophylaxis with oral anticoagulants. However, it is noted that guidelines are not always followed. We sought to learn how Saudi Arabian general practitioners (GPs) self-reported using risk assessment tools and atrial fibrillation clinical practice guidelines created by cardiology associations, as well as how GPs felt about the resources that were available. Through the use of a self-administered questionnaire, we carried out a cross-sectional survey. A total of two-hundred GPs participated in the study. The guidelines were frequently used when a clinical decision regarding anticoagulation therapy appeared difficult (n = 57, 28.4%). The most predominant strengths of participants’ chosen clinical guidelines were clear recommendations (n = 56, 27.9%), easy-to-follow algorithms (n = 39, 16.9%), detailed recommendations supported by evidence (n = 34, 16.9%), and online availability (n = 27, 13.4%). Many respondents said they used a formal stroke risk assessment tool in addition to their clinical judgment as a GP for most decisions (60%). Most respondents preferred using the CHA2DS2-VASc (n = 106, 52.7%), CHA2DS2-VA (n = 45, 22.4%), CHADS2 (n = 35, 17.4%), and GARFIELD (n = 14, 7.0%). HAS-BLED (n = 100, 49.8%) and HEMORR2HAGES (n = 50, 24.9%) were the most frequently utilized formal tools for assessing the risk of bleeding among GPs. Over half of the participants referred to guidelines when deciding thromboprophylaxis in patients with atrial fibrillation. Additionally, many respondents used formal procedures for assessing the risks of bleeding and stroke in addition to their clinical judgement in their roles as GPs. The guideline was assessed as being extremely helpful overall by GPs who used it to make thromboprophylaxis decisions.

## 1. Introduction

A typical cardiac arrhythmia that is often encountered in medical practice is atrial fibrillation (AF) [1]. It affects around 38 million individuals worldwide and is associated with a higher incidence of stroke and systematic embolism [2]. The likelihood of having a stroke correlated with AF predominantly depends on the age of the patient and other prevalent concomitant illnesses [3,4,5]. The risk of stroke is five times higher in patients with AF [6]. 

Patients with AF frequently utilize oral anticoagulants (OACs) for the treatment and prevention of thromboembolic disorders, especially stroke. Similarly, OACs reduce the risk of stroke by 70% [3]. OAC thromboprophylaxis is advised in individuals with moderate- to high-risk stroke according to worldwide cardiology society clinical recommendations [3]. Moreover, guidelines recommend assessing the potential for bleeding with the objective of determining and addressing any bleeding risk factors that can be altered [3,4,5]. 

Thromboprophylaxis is correlated with better treatment outcomes when it follows guidelines that are based on widely used thromboembolic risk assessment tools such as the CHA2DS2-VASc score [7]. Nonetheless, deviations from these recommendations, predominantly undertreatment, are noted [8]. In Australia, evidence indicates that between 19% and 37% of hospitalized patients with AF at higher risk of stroke did not consume OACs [9,10], while 35–45% of high-risk patients in general practice were not prescribed an oral anticoagulant [11,12]. 

OAC undertreatment is common in AF patients for a variety of reasons. Most of the evidence currently available about the causes of undertreatment of thromboprophylaxis in patients with AF is based on retrospective studies that were conducted outside of the general practice environment. [7,10,13,14,15]. However, a prior study found that prescriber-related characteristics, such as their practices and attitudes, were among the major factors [8]. Previous research showed that, despite general practitioners’ (GPs) limited use of bleeding risk assessment tools, focus is placed more on preventing bleeding than stroke when OACs are prescribed. [16,17,18]. 

Heidbuchel and colleagues identified major gaps in knowledge and skills of prescribers treating patients with AF, particularly in calculating and implementing bleeding risk assessment [19]. Another multicenter European study reports that prescribers apply various national and international guidelines for managing individuals with AF; however, twenty-one percent of the study participants did not follow any specific guidelines [20]. A semistructured interview study among GPs in Australia revealed that the decision-making process is a major cause for deviations from thromboprophylaxis guidelines. In addition, it has been shown that additional variables, including frailty, advanced age, and the risk of falling, might affect choices that vary from the thromboprophylaxis advised by guidelines.

The Ministry of Health of the Kingdom of Saudi Arabia (KSA) released clinical practice recommendations for treating patients with AF using antithrombotic medication. [21] However, GPs typically use international recommendations. There is not much research to support where GPs (also known as primary care physicians) in the KSA find information about thromboprophylaxis in AF and how various factors affect GPs’ decision making. Therefore, we aim to ascertain the self-reported utilization of AF clinical practice guidelines created by cardiology societies and risk assessment tools by GPs working in the KSA, as well as the perceptions of GPs towards the available resources.

## 2. Materials and Methods

### 2.1. Study Design and Setting

We conducted an online cross-sectional survey from July 2022 to August 2022 among GPs working in Riyadh, KSA. GPs start their medical practice after completing a bachelor’s degree in medicine from a medical school and medical internship. GPs in the KSA mostly work in community healthcare centers and primary care facilities. [22]

### 2.2. Inclusion and Exclusion Criteria

GPs working in at least one general practice setting in the KSA were recruited for this study.

### 2.3. Data Collection

GPs’ anonymous information was gathered using SurveyMonkey^®^, an online survey management platform. A 16-item self-administered survey was given to GPs working in a general practice setting. This questionnaire was adapted from a previous study that showed acceptable validity and reliability [23]. The face validity and useability of the study tool were also ascertained through a pilot sample of 10 GPs working in the KSA. The study tool was also reviewed to make sure it is simple to understand and complete based on piloting input. The questionnaire collected information about general practitioners’ self-reported use of clinical guidelines and tools for risk stratification in AF as well as sociodemographic information. According to our definition, clinical guidelines are those that are specifically for AF, include recommendations regarding thromboprophylaxis, and are compiled by relevant cardiology societies such as the National Heart Foundation of Australia (NHFA), the Cardiac Society of Australia and New Zealand (CSANZ), the European Society of Cardiology (ESC), and the American Heart Association (AHA)/American College of Cardiology (ACC)/Heart Rhythm Society (HRS); these guidelines are referred to hereafter as ‘clinical guidelines’. Multiple approaches were followed to recruit the GPs, including advertising through professional websites and social media (Twitter, Facebook, and LinkedIn) and via direct contact through medical and professional organizations. 

### 2.4. Ethical Considerations

All participants were briefed about the purpose of the study and provided a study information sheet. A waiver of written informed consent was granted by the ethics committee. The study was initiated after receiving institutional review board ethical permission, and it was carried out in accordance with the Declaration of Helsinki’s principles. In publishing this study, we adhered to the Strengthening the Reporting of Observational Studies in Epidemiology (STROBE) declaration. [24]. 

### 2.5. Sample Size and Statistical Analysis

Taking into account their clinical judgement as a GP, 57% of respondents employed a structured stroke risk assessment technique based on literature findings [23]. Given this proportion, a 95% confidence level, and a 5% margin of error, a sample size of 370 was estimated using an online calculator [25]. The study’s results were summarized using descriptive statistics. Categorical data were expressed as frequencies and percentages, whereas continuous variables were expressed as the median and interquartile range (IQR). The primary (i.e., either “entirely” or “mainly”) reliance on formal stroke risk assessment methods and the primary (i.e., either “entirely” or “mainly”) reliance on formal bleeding risk assessment tools were compared using χ^2^. All analyses were declared significant at *p* < 0.05. All statistical analyses were performed using R^®^ (version 4.0.5).

## 3. Results

A total of 200 individuals participated in the study, with a median (IQR) of 6.5 (33) years of GP practice. The majority, 67 (33.3%), of respondents were younger than 30 years and male (120, 59.7%). Over half of the subjects, 117 (58.2%), had a bachelor’s degree level of education. The sociodemographic details of the participants are shown in Table 1.

Of the 200 participants, 112 (55.7%) used clinical guidelines directly to gather knowledge to inform thromboprophylaxis decisions in patients with AF. Other sources of this information reported by the respondents included educational sessions (30, 14.9%), online continuing professional development (CPD) (35, 17.4%), and reading scientific articles (23, 1.4%). When a clinical decision on anticoagulant therapy appeared difficult, 57 (28.4%) GPs used guidelines the most. A guideline was frequently used when treating individuals who had recently been diagnosed with AF (56, 27.9%) and when a new version was released (44, 21.9%). However, the lowest (43, 21.4%) proportion of the GPs said they referred to guidelines every time they manage patients with AF. When participants were asked about the reasons for not using the guidelines, 70 (34.8%) said it was because of too many guidelines to choose from. Similarly, 52 (24.9%) responded that it was due to too many guidelines for different diseases, while 33 (16.4%) mentioned that the guidelines take a long time to read and consult. Table 2 shows the summary of participants’ use of thromboprophylaxis guidelines. 

The most predominant identified strengths of participants' preferred guidelines were clear recommendations (56, 27.9%), easy-to-follow algorithms (39, 16.9%), detailed recommendations supported by evidence (34, 16.9%), and online availability (27, 13.4%). In contrast, the major limitations highlighted by the respondents were too-long guidelines (38, 18.9%), difficulty in accessing/not user-friendly (31, 15.4%), and limited clinical flexibility or not patient-specific (28, 13.9%). Further, the helpfulness of the clinical guidelines in challenging/uncertain clinical decisions was investigated. Of the total respondents, 70 (34.8%) rated the guidelines as very helpful and 98 (48.8%) as helpful. The strengths and limitations of commonly followed guidelines for thromboprophylaxis are listed in Appendix A Table A1.

**Figure 1:**
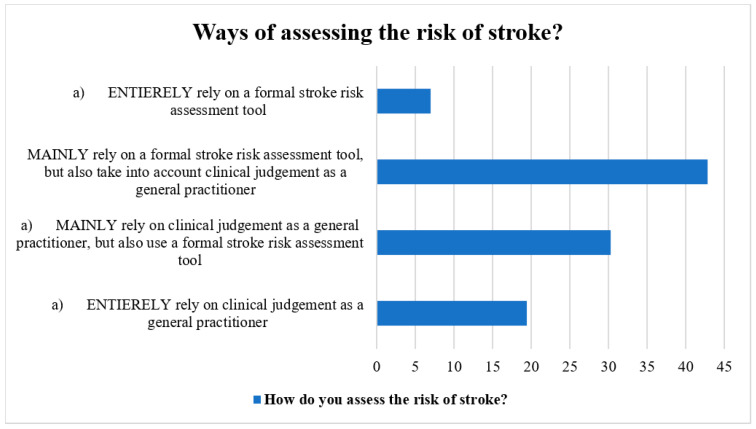
Ways of assessing stroke risk by the participants.

The methods for determining stroke risk in patients with AF were discussed by the participants. Most respondents (40%) utilized a formal stroke risk assessment tool in addition to their clinical judgement as general practitioners, whereas the next greatest percentage (37.5%) used both the official stroke risk assessment tool and their clinical judgement as general practitioners. Additionally, general practitioners stated totally depending on their clinical judgement (17.0%) and employing a structured stroke risk assessment tool (7.0%) (Figure 1).

Regarding the utilization of official stroke risk assessment instruments, most of the respondents preferred using the CHA2DS2-VASc (106, 52.7%), CHA2DS2-VA (45, 22.4%), CHADS2 (35, 17.4%), and GARFIELD (14, 7.0%). These tools were mostly used as part of a regular review (50, 24.9%); anytime a patient’s comorbidities alter (45, 22.4%); and when newly initiating patients on oral anticoagulation therapy (41, 20.4%) (Table 3). Participants’ usage of bleeding risk assessment was also investigated. HAS-BLED and HEMORR2HAGES were the most frequently utilized formal tools among the GPs, reported by 100 (49.8%) and 50 (24.9%), respectively. Another popular tool among the participants was ATRIA (37, 18.4%) (Table 3). The frequency of using the most preferred tool (HAS-BLED) was assessed among the respondents. Most of the respondents used it when newly initiating patients on oral anticoagulation therapy (69, 34.3%), whenever a patient’s comorbidities change (57, 28.4%), and as part of a regular review (50, 24.9%). When participants’ primary use of formal stroke risk assessment tools and primary use of formal bleeding risk assessment tools were compared, there was a significant correlation between the two. This indicated that participants who relied on formal stroke risk assessment tools primarily were more likely to also rely on formal bleeding risk assessment tools (χ^2^ = 42.55, df = 9, *p* < 0.000).

**Figure 2:**
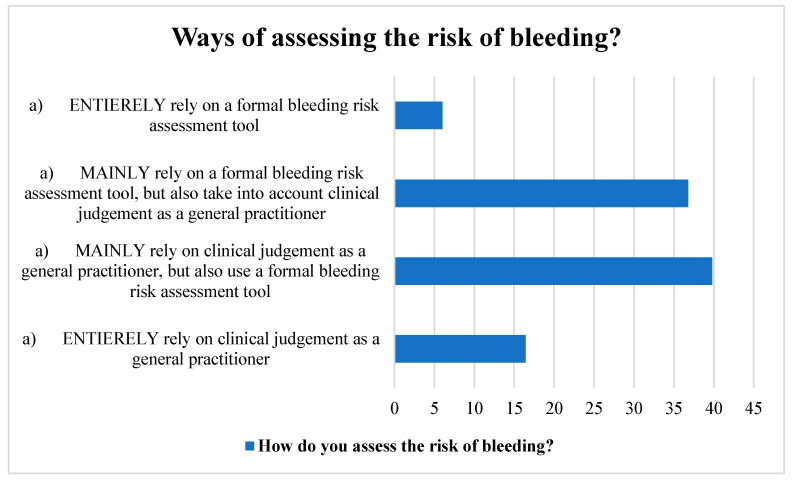
Ways of assessing stroke risk by the participants.

Many participants used a structured bleeding risk assessment method in addition to their clinical judgement as GPs. Likewise, a sizable number of the respondents relied mostly on a formal bleeding risk assessment method, but they also took their clinical judgement into account. A small portion of the participants, however, relied solely on their clinical judgement and a formal instrument for assessing bleeding risk (Figure 2).

## 4. Discussion

Our study was the first to provide comprehensive evidence about the sources where GPs in the KSA obtained information to make judgements about prescribing thromboprophylaxis to patients with an AF diagnosis. Although our participants mentioned other sources of information that guided thromboprophylaxis decisions, over half of the participants in this study referred to guidelines when making thromboprophylaxis decisions in patients with AF. Additionally, most of the participants used their clinical judgement as a GP in accordance with general clinical recommendations for the prescription of OACs, as well as formal bleeding stroke and risk assessment methods. Overall, the respondents rated the routine use of guidelines as very helpful in making thromboprophylaxis decisions. In contrast, the most predominant reason for deviating from guidelines was “too many guidelines to choose from and for different disease conditions". Our findings could improve the utilization of clinical guidelines among GPs in KSA. It can also guide educational intervention for decreasing the rate of deviation from guidelines and recommendations guiding clinical practice. 

In this study, a guideline was most frequently used when a clinical choice on anticoagulant therapy seemed difficult. When starting patients on OACs for the first time, a significant proportion of the participants also used tools to assess the risk of stroke and bleeding. While it is commendable that GPs in our study referred to guidelines and formal stroke and bleeding risk tools for some groups of patients with AF, it may be concerning that other categories of patients may be at higher risk of adverse outcomes given the heterogenous nature of people with AF [26]. The majority of these patients have a variety of clinical factors that could increase the risk of bleeding and other negative outcomes [27]. For example, a study among Asian patients with AF has shown that a deviation from guideline recommendations (undertreatment or overtreatment with OACs) is associated with an increased risk of adverse outcomes when compared with guideline-compliant management [28]. Further, with the advance in age and occurrence of other comorbidities, the stroke and bleeding risks of patients with AF also increase. Thus, the risk assessments of these individuals keep changing and posing a risk of OAC suboptimal management. Therefore, since adherence to OAC management guidelines has been demonstrated to enhance clinical outcomes in patients with AF [28,29], the need to use guidelines across all patients with AF should be promoted among the GPs in the KSA. 

The reasons for deviation from AF guideline recommendations were accessed among the participants. About 44% of the GPs do not use guidelines as a guide to thromboprophylaxis decisions in patients with AF, and the major reason reported was the availability of too many guidelines to choose from. A similar challenge was encountered among GPs in Australia [23], where multiple AF guidelines posed a barrier to thromboprophylaxis management. While future studies are needed to investigate the conflicting areas among the different guidelines, one possible reason may be the recommendation of different stroke risk assessment tools. For example, most AF guidelines such as the 2020 European Society of Cardiology (ESC) [4] and 2018 Korean and 2019 American clinical guidelines recommend using CHA2DS2-VASc stroke assessment tools to guide decisions for thromboprophylaxis [5,30]. However, the 2018 Australian AF guidelines recommend the sexless (CHA2DS2-VA) stroke assessment tool [3]. While the debate on the use of the most appropriate stroke risk scale continues in the literature [31], GPs should always identify and use the most suitable AF guideline in their clinical practice. Additionally, since the Saudi Heart Association (SHA) is an affiliated member of the ESC, the use of this society’s recommended stroke risk scale (CHA2DS2-VASc) should be promoted among the GPs in the KSA. Alternatively, the SHA may consider developing an AF guideline specific to the Saudi population, as with other similar disorders, for example, the Saudi Clinical Practice Guideline for the treatment of venous thromboembolism [32]. 

Regarding deviation from guideline recommendations, some of the participants in this study described the guidelines as very long and time consuming to consult. This challenge may explain the reason why some of the GPs obtain information from other sources. Although the literature suggests developing setting-specific guidelines that are shorter (compared to lengthy guidelines) [23], more education and awareness campaigns on the benefits of adherence to guidelines are needed to improve optimal patient outcomes in the KSA. 

This study is limited by a small sample size (lower than the calculated number) and the online nature of participant recruitment. Although these shortcomings may prevent the generalization of our findings to all GPs in KSA, our study identified sources where GPs in the KSA obtain information about thromboprophylaxis in patients with AF. The study also reported barriers to adherence to AF guidelines that could guide interventional measures for improving guideline use in clinical practice. Future research could address the challenges associated with the implementation of individual guidelines. 

## 5. Conclusions

Over half of the participants in this study referred to guidelines when deciding thromboprophylaxis in patients with AF. Additionally, many respondents used formal procedures for assessing the risks of bleeding and stroke in addition to their clinical judgement in their roles as GPs. The guideline was assessed as being extremely helpful overall by GPs who used it to make thromboprophylaxis decisions. However, the common reason for deviating from guidelines was “too many guidelines to choose from”. Our findings could guide interventions for improving the use of clinical guidelines and subsequent optimal outcomes. There is also a need to ascertain the prescribing practices of GPs in the management of AF as most of them refer patients to specialists.

## Figures and Tables

**Table 1:** Characteristics of the participants (n = 200).

Variable	Frequency (%)
**Age Category**	
<30 years	67 (33.3)
30–39 years	64 (31.8)
40–49 years	46 (22.9)
50–59 years	20 (10.0)
60–70 years	3 (1.5)
>70 years	0 (0.0)
**Gender**	
Male	120 (59.7)
Female	80 (39.8)
**Educational level**	
Bachelor’s degree	117 (58.2)
Master’s degree	57 (28.4)
PhD degree	26 (12.9)

**Table 2:** Participants’ use of thromboprophylaxis guidelines (n = 200).

Variable	Frequency (%)
**Source of information to guide thromboprophylaxis decisions in AF**	
Directly through clinical guidelines	112(55.7)
Educational sessions (e.g., webinars)	30(14.9)
Online CME/CPD websites (e.g., Center for Consulting and Health Skills Training)	35(17.4)
Reading of the scientific literature	23(11.4)
**Frequency of using a guideline**	
When managing patients newly diagnosed with AF	56(27.9%)
When a clinical decision about anticoagulation is challenging or uncertain	57(28.4%)
When a new version of the guideline is available	44(21.9%)
Every time I manage a patient with AF	43(21.4%)
**Reasons for not using AF clinical guidelines as a primary resource**	
Too many guidelines to choose from	50(24.9)
Too many guidelines for different disease conditions	70(34.8)
The guidelines are very long and time-consuming	33(16.4)
The guidelines sometimes disagree with each other	24(11.9)
The guidelines sometimes disagree with PBS criteria	8(4.0)
My busy schedule	4(2.0)
Preference/better familiarity with other options (e.g., GARFIELD tool)	11(5.5)

AF, atrial fibrillation; CPD, continuous professional development; GARFIELD, The Global Anticoagulant Registry in the Field; PBS, Pharmaceutical Benefits Scheme.

**Table 3:** Utilization of stroke and bleeding risk assessment tools by GPs (n = 200).

Variables	Frequency (%)
**Preferred formal stroke risk assessment tool**	
CHA_2_DS_2_-VASc	106(52.7%)
CHA2DS_2_-VA	45(22.4%)
CHADS_2_	35(17.4%)
GARFIELD	14(7.0%)
**Frequency of using the preferred formal bleeding risk assessment tool**	
As part of a regular review (e.g., every 6–12 months)	33 (16.4%)
Whenever a patient’s comorbidities change	57 (28.4%)
When newly initiating patients on oral anticoagulation therapy	69 (34.3%)
Every time a patient has a new medication prescribed	24 (11.9%)
Every time the patient visits my office	7 (3.5%)
Others	10 (5.0%)

## Data Availability

Not applicable.

## Appendix A

**Table A1:** Strengths and limitations of thromboprophylaxis recommendations used frequently in AF (n = 200).

Variable	Frequency (%)
**Strengths of clinical guidelines**	
Clear recommendations	56 (27.9%)
Detailed recommendations supported by evidence	34 (16.9%)
Easy to follow algorithms	39 (19.4%)
Online availability	27 (13.4%)
Clinical applicability/flexibility	22 (10.9%)
Concise	22 (10.9%)
Most authoritative guideline in Australia	0
**Major limitations of clinical guidelines**	
I have not noticed any major limitations.	68 (33.8%)
Too long	38 (18.9%)
Difficult to access/not user-friendly	31 (15.4%)
Do not consider patient preferences	16 (8.0%)
Limited clinical flexibility (not patient-specific)	28 (13.9%)
Unclear recommendations	4 (2.0%)
Difficult to follow algorithms	15 (7.5%)
**Helpfulness of clinical guidelines in challenging/ uncertain clinical decisions**	
Very helpful	70 (34.8%)
Helpful	98 (48.8%)
Slightly helpful	29 (14.4%)
Not helpful at all	3 (1.5%)
